# Nuclear import of Avian Sarcoma Virus integrase is facilitated by host cell factors

**DOI:** 10.1186/1742-4690-5-73

**Published:** 2008-08-07

**Authors:** Mark D Andrake, Monica M Sauter, Kim Boland, Andrew D Goldstein, Maryem Hussein, Anna Marie Skalka

**Affiliations:** 1 Institute for Cancer Research, Fox Chase Cancer Center, Philadelphia, PA 19111, USA

## Abstract

**Background:**

Integration of retroviral DNA into the host cell genome is an obligatory step in the virus life cycle. In previous reports we identified a sequence (amino acids 201–236) in the linker region between the catalytic core and C-terminal domains of the avian sarcoma virus (ASV) integrase protein that functions as a transferable nuclear localization signal (NLS) in mammalian cells. The sequence is distinct from all known NLSs but, like many, contains basic residues that are essential for activity.

**Results:**

Our present studies with digitonin-permeabilized HeLa cells show that nuclear import mediated by the NLS of ASV integrase is an active, saturable, and ATP-dependent process. As expected for transport through nuclear pore complexes, import is blocked by treatment of cells with wheat germ agglutinin. We also show that import of ASV integrase requires soluble cellular factors but does not depend on binding the classical adapter Importin-α. Results from competition studies indicate that ASV integrase relies on one or more of the soluble components that mediate transport of the linker histone H1.

**Conclusion:**

These results are consistent with a role for ASV integrase and cytoplasmic cellular factors in the nuclear import of its viral DNA substrate, and lay the foundation for identification of host cell components that mediate this reaction.

## Background

Integration of viral DNA into the genome of its host cell is an essential step in the replication of all retroviruses. This reaction is catalyzed by the retroviral integrase (IN), an enzyme that, along with reverse transcriptase, enters the cell within the infecting viral capsid. Reverse transcription of the RNA genome to produce retroviral DNA is known to take place in the cytoplasm, shortly after entry. However, the manner in which viral DNA and IN enter the nucleus is not well understood and, indeed, may vary among the different retroviruses. Nuclear import of the human immunodeficiency virus type 1 (HIV-1) preintegration complex, which includes viral DNA and IN, has been the subject of intense investigation. As HIV and other lentiviruses can infect non-dividing cells, in which nuclei remain intact, some nuclear import mechanism must exist for these viruses. In addition to IN, the HIV Gag proteins, matrix (MA) and Vpr, as well as a unique central DNA flap, have been proposed to contribute to this process, although none of the latter three components appear to be essential and details of the process remain controversial and unresolved [1,2]. We and others have shown that the avian sarcoma virus (ASV), an alpharetrovirus, can infect cycle-arrested cells [3,4] and terminally-differentiated neurons [5] quite efficiently. Furthermore, both HIV and ASV can enter the nucleus in cycling cells during interphase, before nuclear disassembly [6,7]. These findings indicate that some mechanism for nuclear import must also be available for ASV.

**Figure 1:**
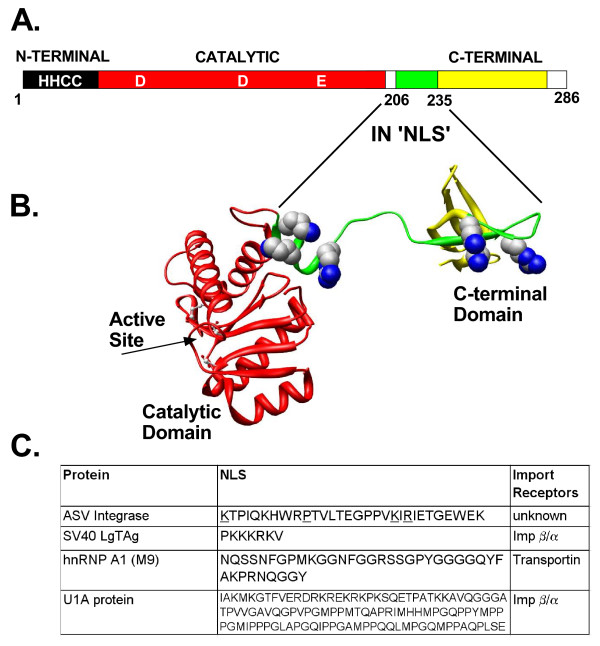
**The ASV IN NLS and three well characterized NLSs**. **A**. Linear map of ASV IN showing the location of NLS sequence. The 286 amino acid IN protein is composed of three domains. The N-terminal, Zn-binding (HHCC) domain (dark) and the central catalytic core domain (red) with the locations of the active site residues (D, D, E) are indicated. The nuclear localization signal, amino acids 206–235 (green), extends from a linker region and into the C-terminal domain (yellow). **B**. A 3-D structural ribbon model of the catalytic core and C-terminal domains of ASV IN [58] with the with basic residues of the NLS shown in space filling representation. Active site residues in the core domain are shown in ball and stick representation. **C**. Comparison of the sequences of the ASV IN NLS with three well-characterized NLSs used in the studies reported herein. Residues underlined in the ASV IN NLS have been shown to be required for function.

Nuclear import occurs through large, multi-protein pore complexes that span the nuclear envelope of eukaryotic cells. Passage through these pores is a multi-step process facilitated by nuclear localization signals (NLSs) that are embedded in import substrates called "cargos." Classical NLSs are characterized by clusters of basic amino acids, and can be grouped into two related categories [8]. The monopartite NLSs, such as that in the SV40 large T antigen (SV40 TAg) (Fig. 1C), contain a short, continuous stretch of basic residues [9,10]. Bipartite NLSs, including the nucleoplasmin NLS [11], contain two clusters of basic residues separated by a spacer region of at least 10 amino acids.

Much of our knowledge of the mechanism of nuclear translocation comes from the study of these model NLSs using an *in vitro *assay that employs digitonin-permeabilized cells [12,13]. In this assay, nuclear import of proteins containing classical NLSs requires a nucleoside triphosphate, ATP or GTP, a functional NLS, and is dependent on the addition of cytosolic extract or purified cytosolic proteins [12]. Studies with this system have led to the purification of two soluble proteins, Importin-α (Impα) [14,15] and Importin-β (Impβ) [16,17], and others [18,19] that participate in import [20] of these NLSs-containing proteins. In the classical pathway, Impα acts as an adaptor protein, binding both to the NLS on the cargo protein and to a specific site on Impβ, which then mediates transport through the nuclear pore complex. In other, non-classical pathways, import is mediated by Impβ alone, or by one or more of a number of other transport receptors and NLSs [21].

Our previous investigations identified a nuclear localization signal in a linker region between the catalytic core and C-terminal domain of ASV IN (Fig. 1). This sequence, comprising 30 amino acids (residues 206–235), is sufficient to target a cytoplasmic protein to the nucleus of mammalian cells in transient transfection assays [22]. We have also observed that substitution of specific Lys or Arg residues within this sequence had no effect on the activities of the purified ASV IN proteins *in vitro*, but prevented nuclear accumulation of a Lac-fusion construct and caused delayed replication kinetics when the corresponding mutations were included in the viral genome [23]. Subsequent studies have shown that the IN domain of the β subunit in the ASV heterodimeric reverse transcriptase (RT) accounts for its nuclear accumulation when expressed independently [24]. As integrase is a component of the functional ASV pre-integration complex, we have proposed that this protein may facilitate nuclear transport of the viral DNA to which it is bound. Because the NLS of ASV IN has only limited similarity to the mono- or bi-partite classical NLSs [20], and no similarity to several other known NLSs (Fig. 1C), it seemed possible that this sequence represents a distinct class of karyophilic signals. Here we describe studies of the nuclear import of the ASV IN protein using *in vitro *assays with digitonin-permeabilized cells [12], and investigate whether such import exploits the classical transport receptors.

## Results

### The NLS of ASV integrase mediates nuclear transport of a cytoplasmic protein

**Figure 2:**
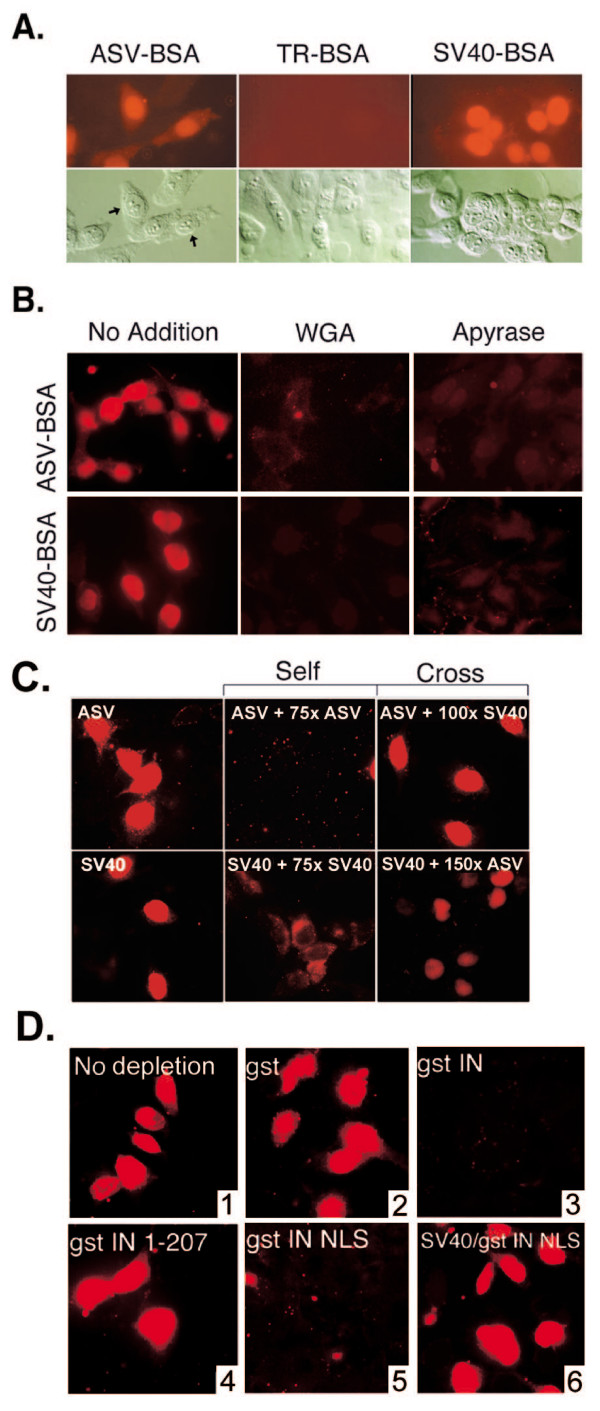
**Nuclear import of ASV-BSA and SV40-BSA substrates; import of ASV-BSA does not require the Impα-Impβ pathway**. **A**. Digitonin-permeabilized HeLa cells were incubated in the presence of complete transport mixture containing the ASV-BSA conjugate, the SV40-BSA conjugate, or Texas red-labeled BSA (TR-BSA). Top panels: Visualization of Texas red conjugates by fluorescence microscopy. Bottom panels: Differential interference contrast (DIC) microscopy of the same field to show preservation of cell integrity. **B**. Digitonin permeabilized HeLa cells were untreated (no addition), treated with 50 μg/ml wheat germ agglutinin (WGA), or 50 units/ml apyrase (Apyrase) prior to incubation with complete transport mixture containing either the ASV-BSA or the SV40-BSA import substrates. **C**. Free NLS peptides were added to the import reactions in molar excess of the import substrates as indicated. "Self" signifies competition with the homologous peptides; "Cross" indicates competition for ASV-BSA import by excess SV40TAg NLS peptide or competition for SV40-BSA import by excess ASV NLS peptide. The left column panels show import in the absence of competitor peptides. **D**. Depletion of ASV-BSA import factor(s) from cytosolic extracts. All assays included Texas-Red labeled ASV-BSA except that shown in the lower left hand corner (panel 4) which included Texas-Red labeled SV40-BSA. Cytosol was either not treated (1; no depletion) or pretreated with glutathione-beads that bound GST alone (2) or fusion proteins of GST plus IN(1–207) which lacks the IN NLS (3), full-length IN(1–286) (5), or a fragment of IN(201–236) that contains the IN NLS (panels 4 and 6).

To determine if the NLS of ASV IN can function in the *in vitro *nuclear import assay we used HeLa cells [12], which are known to support the early steps in replication of a number of retroviruses, including ASV. A traceable import substrate was prepared by crosslinking a peptide comprising the 30 amino acid NLS to Texas red-labeled bovine serum albumin (hereafter called ASV-BSA). As a positive control, a peptide corresponding to the well-characterized, classical karyophilic signal of SV40 Large T antigen [10] was also crosslinked to Texas red-labeled BSA (SV40-BSA). HeLa cells were treated with digitonin to permeabilize the plasma membrane to passage of macromolecules while leaving the nuclear membrane intact, and import assays were performed as described by Adam *et al*. [12]. A HeLa cell cytosolic extract was added to provide any essential components that were lost during permeabilization. Subsequent inspection of these cells by fluorescence microscopy revealed that the ASV-BSA conjugate accumulated in the nuclei (Fig. 2A; top, left panel), whereas there was no nuclear accumulation in cells incubated in the presence of Texas red-labeled BSA alone (TR-BSA) (Fig. 2A; top, middle panel). The latter result was expected, as a molecule the size of BSA (68 kDa) is too large to enter the nucleus by passive diffusion [25]. The SV40-BSA conjugate also accumulated in the nuclei of the permeabilized cells, as was anticipated from previous reports [12] (Fig. 2A; top, right panel). To verify that the nuclear membrane remained intact under our experimental conditions, the cells were incubated in the presence of an antibody to the cytosolic hnRNP protein A1 following digitonin treatment. No nuclear staining of A1 was apparent (data not shown), confirming that the nuclear envelope was not permeabilized by this treatment.

The lectin wheat germ agglutinin (WGA) binds specifically to O-linked N-acetylglucosamine residues, a modification found on many nuclear pore complex proteins [26]. Previous studies have demonstrated that import through the nuclear pore is blocked by WGA both *in vitro *and *in vivo *[27,28]. To determine if WGA inhibits nuclear import of ASV-BSA, permeabilized cells were treated with WGA for 20 min at 20°C prior to incubation in complete transport mixture without added lectin. As shown in Fig. 2B (middle panels), nuclear import mediated by both the ASV IN NLS and the SV40 T Ag NLS was inhibited by WGA, providing evidence that the corresponding conjugates enter the nucleus through the nuclear pore complexes.

To determine if import mediated by the ASV IN NLS requires ATP, the digitonin-treated HeLa cells were pretreated with apyrase to deplete residual ATP. Cells were then incubated in complete transport mixture supplemented with the same concentration of apyrase for 30 min at 30°C. As seen in Fig. 2B (right panels), apyrase treatment reduced the nuclear accumulation of both the ASV-BSA and SV40-BSA transport substrates. In addition, no nuclear import was observed when the transport reactions were performed at 4°C (data not shown). Collectively, results from these experiments indicate that the ASV IN protein contains an NLS that can mediate import of a large cytoplasmic molecule through nuclear pore complexes in a temperature-dependent manner, and that this transport requires ATP or another nucleotide that is dependent on ATP for regeneration [29,30].

### Nuclear import of the ASV-BSA conjugate is saturable and requires soluble cytosolic factor(s), but utilizes a pathway distinct from that of SV40-T-Antigen

Protein import to the nucleus is a signal-mediated process that exhibits saturation kinetics, which reflect the finite amounts of transport receptors available for a given cargo [31]. To determine if import of ASV-BSA can be saturated in our *in vitro *assay, increasing amounts of free ASV IN NLS peptide were added to the nuclear import reactions. Results summarized in Fig. 2C (top, labeled Self) show that addition of a 75-fold molar excess of the free peptide was sufficient to completely inhibit nuclear accumulation of ASV-BSA.

Although longer than the classical SV40TAg NLS, the ASV NLS contains at least three basic amino acids that are critical for nuclear accumulation [[23], underlined in Fig. 1C]. To determine if the ASV IN NLS and the SV40 TAg NLS interact with the same cytosolic NLS binding protein, excess free SV40 TAg NLS peptide was added to the import reactions. The results showed that although addition of excess SV40 TAg NLS peptide blocked the SV40-BSA import reaction (Fig. 2C bottom, Self), addition of an equivalent or even higher (100-fold) molar excess of this peptide had no effect on nuclear import of the ASV-BSA conjugate (Fig. 2C top, labeled Cross). Furthermore, equivalent or higher (150-fold) molar excess of the ASV IN NLS peptide failed to block import of the SV40-BSA conjugate (Fig. 2C bottom, Cross). These data strongly suggest that Impα, the cytosolic adaptor known to bind the NLS of SV40 TAg is not required for import of the ASV IN NLS.

Importins are soluble transport receptors that bind to NLS-containing cargo proteins in the cytoplasm [8]. However, some proteins do not require such receptors for nuclear transport. In these cases, import many be mediated through direct interactions with components of the nuclear pore complex [32,33]. To determine if ASV IN NLS import is dependent on a soluble factor(s) present in the HeLa cytosolic extract, cellular proteins that bind to IN were depleted from these extracts by treatment with immobilized glutathione-S-transferase (GST)-fusion proteins that contained all, or specific segments of IN. No import of the ASV-BSA conjugate was detected after depletion with the fusion protein that contains full length IN (GST-IN (1–286)), or the isolated IN NLS (GST-IN(201–236)) (Fig. 2D, panels 3 and 5). On the other hand, depletion with the latter protein did not affect the ability of the extract to support nuclear import of the SV40-BSA conjugate (Fig. 2D, panel 6). Depletion of the extract with GST-beads alone or with GST-IN(1–207) that lacks the IN NLS, had no effect on the nuclear import of ASV-BSA (Fig. 2D, panels 2 and 4).

The results in Fig. 2 confirm that the ASV-BSA conjugate cannot pass through the nuclear pore unassisted, but rather that soluble cytosolic factor(s), necessary for nuclear import, bind specifically to the ASV IN NLS to facilitate its transport. The data also confirm that the cytosolic component(s) that binds the ASV IN NLS to facilitate nuclear transport is distinct from that which binds SV40-BSA.

### ASV IN does not compete for factors required for SV40 TAg or U1A NLS-mediated import

**Figure 3:**
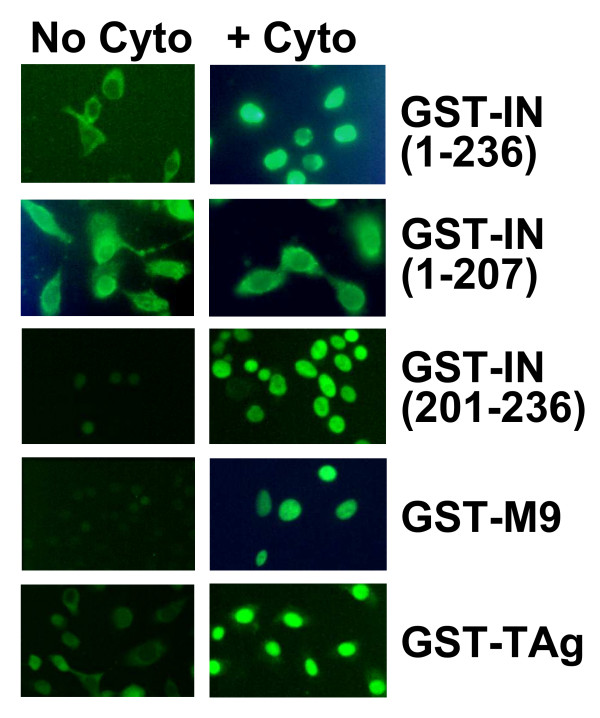
**Nuclear import of GST-NLS substrates in digitonin-permeabilized HeLa cells**. GST-NLS fusion proteins were incubated in digitonin permeabilized HeLa cells for 30 min at 37°C prior to fixation with paraformaldehyde and staining with fluorescent antibody against GST. Left column panels are import without added cytosol and right column panels with added HeLa cytosol extracts.

The studies described above were designed to monitor the activity of the isolated NLS of ASV IN in comparison to the classical NLS of SV40 TAg. To compare the properties of IN NLS-mediated import with those of other characterized but unusual classes of NLSs (Fig. 1C), we prepared GST-fusion proteins that included the full length IN or specific truncated versions of this protein, as well as fusion proteins that included the following: the M9 NLS of hnRNP-A1 protein, which binds the Impβ-related protein, Transportin (GST-M9) [34], the NLS of U1A protein, which mediates import of U1 RNA (GST-U1A) [35,36] and binds Impα, and the SV40 TAg NLS (GST-TAg) [9,10]. Use of a common fusion partner in this and subsequent assays allowed uniform detection by immunofluorescence with a labeled antibody against GST. Results from import assays with each of these purified GST-fusion proteins are summarized in Fig. 3. They show that all of the NLS-containing proteins were imported into HeLa nuclei as expected, and that such import is dependent on the addition of cytosolic extract. In contrast, the fusion protein GST-IN(1–207), which contains the first two domains of IN but not the NLS, was excluded from the nuclei.

**Figure 4:**
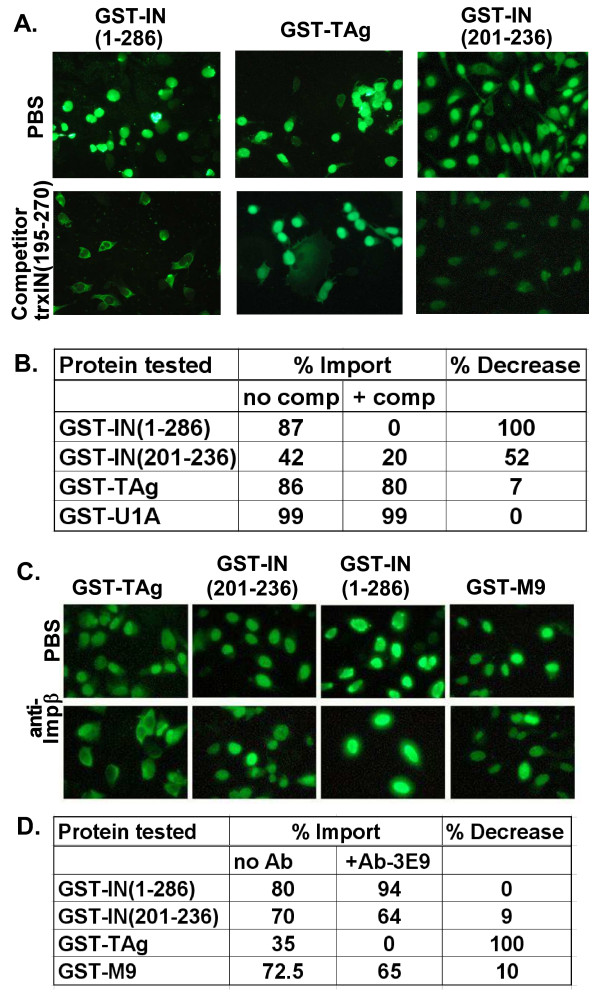
**ASV IN NLS import does not compete for import factors required for SV40-TAg and U1A nuclear accumulation**. **A**. Digitonin permeabilized HeLa cells were either treated with buffer (PBS – top row), or with a molar excess of the competitor protein trxIN(195–270) (bottom row). GST-IN(1–286) and GST-TAg had a 15-fold excess of competitor while GST-IN-NLS(201–236) had a 30-fold molar excess. Import assays were performed as shown in Fig. 3 and staining was done with fluorescent antibody against GST. **B**. Quantitative analysis of nuclear import of various GST fusion proteins with (+ comp) and without (no comp) competitor. More than 100 cells were counted for each experimental condition and the percentage of cells that had a mostly nuclear staining for the fusion protein was calculated. The percent decrease in the presence of the competitor is shown in the column on the right. The lower value for import of GST-IN (201–236) compared to GST-IN (1–286) reflects the fact that a larger percentage of cells had whole cell staining (in which nuclear import could not be assessed) or nuclear exclusion. **C**. Digitonin permeabilized HeLa cells were either treated with buffer (PBS – top row), or with a 50 ug/ml antibody 3E9 against Impβ (bottom row)during the import reaction. **D**. Quantitative analysis of nuclear import of various GST fusion proteins with (+ Ab3E9) and without (no Ab) antibody 3E9. More than 100 cells were counted for each experimental condition and the percentage of cells that had a mostly nuclear staining for the fusion protein was calculated. The percent decrease in the presence of the antibody is shown in the column on the right.

To evaluate the significance of the findings in Fig. 2C and 2D, we next asked if import of the IN fusion proteins shared any of the cytosolic components that are required for import of GST-TAg or GST-U1A. For these studies, a competitor thioredoxin fusion protein was prepared that included the C-terminal domain of ASV IN (residues 195 to 270, which includes the NLS). As shown in Fig. 4A, the presence of a 15-fold molar excess of this IN competitor blocked nuclear accumulation of the full length IN protein (GST-IN(1–286)); only cytoplasmic staining was observed. As expected, nuclear import of the fusion protein containing only the NLS peptide (GST-IN(201–236)) also was decreased upon addition of the competitor, and there was no detectable effect of the competitor on the nuclear accumulation of GST-TAg. Data tabulated in Fig. 4B were obtained by examining the localization of the indicated fusion proteins in more than 100 cells in the absence or presence of the competitor. The results of these analyses indicate that ASV IN NLS-mediated import is distinct from that of both SV40-TAg and U1A NLSs.

As a final test of this hypothesis, a monoclonal antibody (3E9) known to block classical import mediated by Impα/Impβ heterodimer [37] was included in nuclear import assays with the GST-IN proteins. As seen in Fig. 4C, addition of this reagent resulted in exclusion GST-TAg from the nuclei. This result is expected, as import of the SV40 TAg is known to be dependent on formation of a complex between Impα and Impβ. In contrast, the antibody had no significant effect on nuclear accumulation of fusion proteins that included full length IN, a C-terminal fragment of IN containing the NLS or, as expected, GST-M9 (Fig. 4C; compare top and bottom rows). Quantitation of the results of these experiments is summarized in Fig. 4D.

### Nuclear import of ASV IN shares factors required for import of linker histone H1

**Figure 5:**
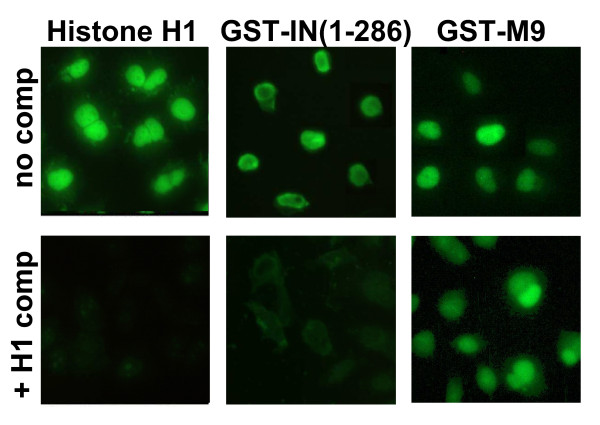
**ASV IN mediated import is inhibited by excess histone H1**. The import of labeled histone H1, (GST-IN(1–286), and the Impβ binding domain fused to GFP (IBB-GFP) was examined in the absence (top) and presence (bottom) of excess unlabeled histone H1. Incubations were for 30 min and all exposure times were equivalent.

Impβ is known to play a role in the nuclear import of several basic, nucleic-acid binding proteins such as histones and ribosomal proteins, but does so using adapter Importins other than Impα [38,39]. As ASV IN is also a basic protein (pI of 9.8), it seemed possible that nuclear import of ASV IN might involve other transport receptors that mediate import of highly basic cellular proteins. To examine this possibility, competition experiments were performed with histone H1. The linker histone H1 appears to depend mainly on the action of an Impβ-Imp7 heterodimer, but other Impβ-like receptors can also mediate its transport [38,39]. As illustrated in Fig. 5, nuclear import of histone H1 is saturable in our assay; nuclear accumulation of the labeled protein was competed by a 15-fold molar excess of unlabeled histone H1. Under these same conditions, import of GST-IN(1–286) was also inhibited by unlabeled histone H1. In contrast, import of the GST-M9, which utilizes a distinct pathway, mediated by Transportin, was unaffected by the competitor. This result shows that the excess histone H1 is not simply blocking all nuclear import, but is a specific competitor for import of ASV IN. While the results with 3E9 antibody in Fig. 4 rules out a role for the Impα/Impβ heterodimer in ASV IN transport, it does not preclude Impβ cooperating with any of several other importins involved in histone import. We conclude, therefore, that ASV IN NLS import requires one or more of the transport receptors utilized by histone H1.

### Two characteristic import rates

**Figure 6:**
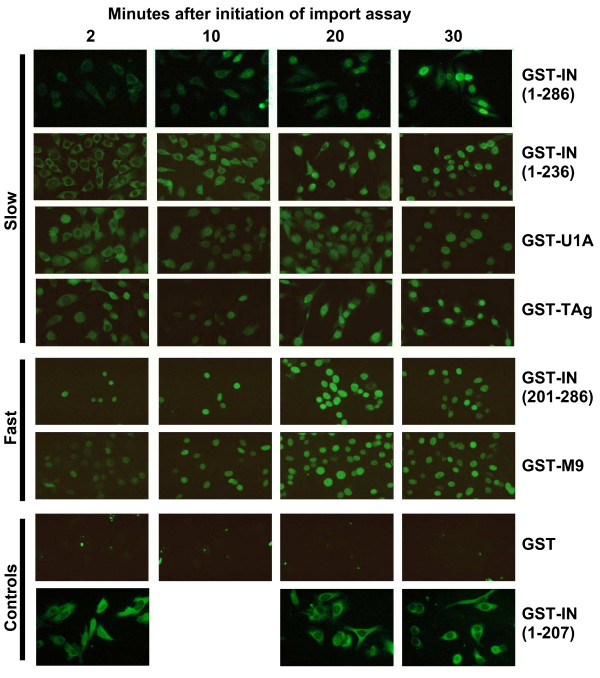
**Kinetics of ASV-NLS mediated import**. GST-NLS fusion proteins were incubated in digitonin permeabilized HeLa cells for various times (labeled above each column) at 37°C prior to fixation with paraformaldehyde and staining with fluorescent antibody against GST. The fusion protein used in each row is labeled at the right and the properties described in the text. Fusion proteins that are imported with slower kinetics are grouped at the top (rows 1–4), and those with faster kinetics in the middle (rows 5 and 6). Control fusion proteins that are not imported into the nucleus are in rows 7 and 8.

During the course of our analyses, we observed variation in the rates of nuclear accumulation with different GST-fusion proteins. To examine these differences more systematically, we monitored nuclear uptake at specified times subsequent to initiating the import reaction (Fig. 6). We observed that these proteins fell into two categories. Fusion proteins that contain full-length IN, C-terminally truncated IN, or the UIA or SV40TAg NLSs, accumulated in the nuclei slowly, and the proteins initially appeared to be retained within the cytoplasmic compartment of the permeabilized cells. Fusion protein containing the M9 NLS or the isolated IN NLS fragment were found only in the nuclei even at the earliest time points, with nuclear staining increasing over time. Control experiments verified that GST alone does not accumulate in nuclei or the cytoplasm compartment. However, while the fusion protein containing IN that lacked the NLS (GST-IN(1–207)) was excluded from the nucleus as expected, it was retained in the cytoplasmic compartment throughout the period monitored in this assay. Similar phenomena are observed in the absence of ASV IN NLS or SV40 Tag NLS-mediated import in other data presented herein (see Figs. 2C, 3, 4A &4C). From these results we conclude that determinants in the N-terminal and/or catalytic core domains mediate attachment of IN protein to cytoplasmic components of the cell that remain after permeabilization.

## Discussion

The studies reported here exploit an *in vitro*, permeabilized cell assay to investigate the nuclear import of ASV IN, mediated by an NLS initially identified in transient transfection experiments [22,23]. This *in vitro *cell assay makes it possible to monitor nuclear import directly, and to delineate critical properties of the reaction. Use of a large substrate comprising the NLS peptide crosslinked to bovine serum albumin revealed that NLS-mediated import can be blocked by wheat germ agglutinin and is, therefore, dependent on transport through the nuclear pore complex. Such transport was also shown to be saturable, and to require soluble cellular factors. Sensitivity to treatment with apyrase, which could be reversed by addition of ATP, was also observed.

The requirement for ATP could reflect a need for replenishment of GTP. The GTP-bound form of the Ran GTPase is concentrated in the nucleus, where it binds to importins and causes release of their cargo. Depletion of ATP, with concomitant decrease in Ran GTP, is known to decrease the recycling of importins to the cytoplasm [40,41]. However, recycling of import receptors may not be required in the permeabilized cell assay if an excess of the relevant Importin is present in the cytosolic extract. Therefore, it is also possible that the ASV IN NLS-mediated import is Ran-GTP-independent and, as is the case for the transit of some large proteins, ATP is required for transit through the nuclear pore complex [42,43]. Further studies will be required to distinguish between these two possibilities.

We have also used this permeabilized cell assay to analyze the nuclear import of fusion proteins containing full length ASV IN or specific segments of this protein. Our results show that the ASV IN NLS is also active within the context of the full protein or segments of the protein that include the NLS. Constructs containing IN segments that lacked the NLS were not imported to the nucleus, indicating determinants essential for nuclear import of IN are contained within the identified NLS. These results are consistent with our previous transfection studies, in which nuclear accumulation of various Lac-IN fusion proteins was monitored [23].

Although the ASV IN NLS comprises an apparently unique sequence, it does bear some similarity to classical bipartite NLSs such as nucleoplasmin, comprising clusters of basic residues separated by a spacer. We therefore considered the possibility that import of ASV IN might depend on the same cellular factors that mediate import of the classical NLSs, the adapter Impα and Impβ. This hypothesis was tested in a variety of ways. Competition experiments with the BSA conjugates showed that addition of excess amounts of peptides corresponding to the classical SV40 TAg NLS or the IN NLS could block nuclear import mediated by the corresponding NLS, but had no effect on the activity of the other. We also found that excess IN NLS did not compete for nuclear import mediated by the U1A NLS, even though IN- or IN NLS-mediated import was abolished. Lastly an antibody that blocks Impα/Impβ mediated SV40 T-antigen import was not observed to inhibit ASV IN import. All these experiments failed to support the hypothesis that transport of ASV IN requires this classical pathway. We concluded from these results the ASV IN NLS does not bind Impα nor utilize the Impα/Impβ heterodimer.

Basic residues are also known to be critical for binding to Impβ by various nonclassical NLS sequences that, like the ASV IN NLS, are Impα-independent. For example, structural analyses of the parathyroid hormone-related protein (PTHrP) NLS bound to Impβ reveal a requirement for a cluster of basic amino acids followed by a twist in the peptide and then an extended segment. This NLS binding is stabilized by a combination of charge interactions with the basic residues and hydrophobic interactions with the extended peptide [44]. As several basic residues as well as one proline are required for IN NLS function [23], both its conformation and accessibility (see Fig. 1) are consistent with this type of interaction, and it remains conceivable that the soluble cellular factor(s) required for ASV IN import is a β-like Importin [21] acting alone or in conjunction with Impβ.

ASV IN is a highly basic protein (pI of 9.8), and excess histone H1 competes for ASV IN import in our assay. While H1 is best transported by the Impβ/Imp7 heterodimer it has been shown to bind to Imp5, as well as Impβ or Imp7 alone. The core histones are even more promiscuous in their usage of various importins [45,46], as are several other proteins such as c-Jun [47], and other viral proteins (Rev) [48]. As noted below, this also seems to be the case for HIV IN, for which several import pathways have been identified. An excess of histone H1 might then be expected to sequester several other importins in addition to the Impβ/Imp7 heterodimer. We speculate that ASV IN may also have the capacity to utilize more than one import receptor, for example, those that mediate the nuclear import of other basic cellular proteins, such as ribosomal proteins and core histones. Several of these are reported to function as cytoplasmic chaperones that prevent polyanion-mediated aggregation of these basic proteins as well as mediators of nuclear import [39]. Our data suggest that ASV IN takes advantage of one or more of the transport pathways for such basic cellular proteins, which are distinct from the classical NLS pathways, but essential for cell metabolism.

In measuring the kinetics of nuclear import in the permeabilized cells, we observed very rapid accumulation (within 2–10 min) with GST-fusion proteins that included the isolated M9 or IN NLS sequences. A different pattern was observed with fusions that included full length IN or IN(1–236), which also contains the NLS. In these cases we observed staining only in the cytoplasmic compartment in the 2–10 min time period, and the fusion proteins were largely excluded from the nuclei. Upon further incubation, for 20–30 min, staining was no longer seen in the cytoplasmic compartment, but the fusion proteins with the IN NLS now localized to the nuclei. This difference could not be attributed to size of the cargo, as the smaller fusion proteins containing only the SV40 TAg NLS or the U1A NLS exhibited the same slow patterns observed with the full length IN protein. Nor is this binding to cytosolic components likely to be due to aggregation; the IN fragment 1–207 is monomeric in solution at high concentrations, and yet this protein exhibits prominent cytoplasmic binding. The simplest explanation of these results is that ASV IN protein and some of the isolated NLSs can bind to cytoplasmic components. The biological significance of this observation is unclear, as soluble components are lost from the permeabilized cells, and cytoskeletal or other remaining components may be exposed in some aberrant fashion. Comparison of the patterns obtained with proteins containing the full length IN or IN(1–236) with IN(201–286) suggest that interaction with these cellular components may retard nuclear uptake. When nuclear import cannot occur due to lack of an NLS, as with GST-IN(1–207), cytoplasmic staining was maintained throughout the course of the experiment. This indicates that determinants responsible for interactions with the cytoplasmic components are contained within the N-terminal and catalytic core domains of the IN.

Investigations of the nuclear import of HIV-1 IN have implicated the classical Impα-Impβ [49] and also Imp7 in this process [50]. Using digitonin-permeabilized cells, Fassati and coworkers [51] (supplementary data) reported that Imp7 promotes nuclear transport of purified HIV-1 reverse transcription complexes (RTCs), and that siRNA-knockdown of Imp7 inhibits HIV-1 infection. These findings are consistent with a model in which the interaction between Imp7 and HIV-1 IN facilitates Impβ nuclear import of the preintegration complex. More recent experiments with this same *in vitro *assay have provided evidence that certain tRNAs may also promote RTC import [52], and the role of another importin in HIV-1 infection, Transportin 3, has been reported [53], further implicating multiple pathways in this process.

As noted above, our results fail to support a role for Impα-Impβ in nuclear transport of ASV IN. In preliminary experiments, using transduction of a reporter gene as a readout for successful nuclear import, we observed that while siRNA knockdown of Imp7 reduced transduction by an HIV-1 vector, it had little effect on transduction by ASV. Differences in import pathways for these two retroviruses are not unexpected. The NLS of ASV IN is not conserved among the retroviral genera, and although reports of the location of NLS sequences in HIV-1 IN vary, residues that bind Imp7 have been identified in the C-terminal, SH3-like domain, distal to the location of the NLS in ASV IN [50]. This suggests that some other member(s) of the Importin superfamily or other karyophilic macromolecules promote import of ASV IN.

As with HIV [1,2], NLSs have been found in ASV Gag proteins. Analysis of the function of these sequences suggest that nuclear entry mediated by the basic NLS in the ASV nucleocapsid (NC) protein requires the classical Impα-Impβ, while import mediated by the more unusual NLS in the matrix protein (MA) is facilitated by other members of the Importin superfamily [54]. It has been proposed that these signals may allow the ASV Gag polyprotein precursor to enter the nucleus and capture viral RNA genomes for virion assembly [55]. The possibility that the mature Gag proteins could also contribute to nuclear import of the preintegration complex has been noted, but the biological role for these Gag NLS sequences remain uncertain. Further study, using the system described here and purified transport receptors should make it possible to identify the specific factors required for nuclear import of ASV IN and to evaluate the role of this viral protein in shepherding viral DNA through the nuclear pore.

## Methods

### Cell culture and antibodies and photomicroscopy

HeLa cells were obtained from the Fox Chase Cell Culture Facility and passaged in DMEM with 10% FCS, 1 unit/ml penicillin and 1 ug/ml streptomycin. Antibody against hnRNP-A1 was a gift from Dr. Gideon Dreyfuss (University of PA). Monoclonal antibody 3E9 was provided by Stephen Adam (Northwestern University). Immunofluorescence microscopy was performed on an Olympus BK2 microscope. Color images were taken with Kodak Ektachrome 400 film or Olympus MagnaFire digital camera, maintaining equal exposure times within each experiment. Glutathione-S-transferase (GST)-fusion proteins used as import substrates were detected by direct immunofluorescence with labeled antibody against GST (rabbit IgG fraction, Alexa Fluor^® ^488 conjugate – Molecular Probes).

### Preparation of labeled BSA import substrates and cytosolic extract

The ASV IN NLS (NH_2_-cgggtKTPIQKHWRPTVLTEGPPVKIRIETGEWEK-COOH) and the SV40 TAg NLS (NH_2_-cgggGPKKKRKVED-COOH) [10] peptides were synthesized by Research Genetics (Huntsville, AL). The lower case letters represent a linker containing three glycine residues and an N-terminal cysteine for coupling to the BSA. High purity bovine serum albumin (BSA) (Sigma) was labeled with Texas red sulfonyl chloride (Pierce) following published procedures [56]. Labeled BSA was activated with the heterobifunctional cross-linker sulfosuccinimidyl-4(maleimidomethyl) cyclohexane-1-carboxylate (Sulfo-SMCC, Pierce) following the manufacturer's recommendation. A 50-fold molar excess of the ASV IN NLS or the SV40 TAg NLS peptide was bound to the activated-labeled BSA. The average number of peptides cross-linked to the labeled BSA was determined by SDS-PAGE for each import substrate.

HeLa cytosolic extract was prepared as described [12] from pellets of exponentially growing HeLa S3 cells obtained from the Cell Culture Center (Minneapolis, MN) of the National Center for Research Resources. The extract was concentrated to yield a final protein concentration of approximately 40 mg/ml, as determined by Bio-Rad protein assay. Extracts were stored in aliquots at -80°C, and diluted 1:1 for import assays.

### Nuclear import assays

HeLa cells were grown on 8-chamber poly-lysine coated culture slides (BD Biocoat) or coverslips coated with 0.2 mg/ml poly-D-lysine (Sigma). Cells were rinsed in cold transport buffer (20 mM Hepes, pH 7.3, 110 mM potassium acetate, 5 mM sodium acetate, 2 mM magnesium acetate, 1 mM EGTA, 2 mM DTT, and 1 μg/ml each aprotinin, leupeptin, and pepstatin) and immersed in the same buffer containing 30 μg/ml digitonin (Calbiochem) for 5 min to permeabilize the plasma membrane. Coverslips were washed twice with cold transport buffer and inverted over a drop of complete transport mixture for 30 min at 30°C. Cells were then washed in cold transport buffer, fixed 10 min at room temperature with 2% paraformaldehyde in PBS, and washed in PBS prior to mounting with Citifluor (UKC Chem. Lab, Canterbury, UK). The complete transport mixture contained 50% cytosolic extract, approximately 10 μg of import substrate, 20 mM Hepes, pH 7.3, 110 mM potassium acetate, 5 mM sodium acetate, 2 mM magnesium acetate, 2 mM DTT, 1 mM EGTA, 2 mM ATP, 2 mm GTP, 5 mM creatine phosphate (Calbiochem), 20 U/ml creatine phosphokinase (Calbiochem), 10 μg/μl unlabeled BSA, and 1 μg/ml each aprotinin, leupeptin, and pepstatin. Using various concentrations of digitonin and an anti-A1 antibody to monitor nuclear breakdown, it was determined that 30 μg/ml digitonin gave optimal (30–50%) nuclear import without significant nuclear destruction. Higher amounts of digitonin produced detectable nuclear breakdown.

For experiments with wheat germ agglutinin (WGA) inhibition, cells were incubated with transport buffer containing 50 μg/ml WGA (Sigma) for 15 min at 20°C, prior to incubation with complete transport mixture without added lectin. For the apyrase experiments, coverslips were pre-treated (10 min at 30°C) in transport buffer supplemented with 50 U/ml apyrase (Sigma), 1 mM CaCl_2_, and 10 μg/μl unlabeled BSA. Coverslips were then incubated in the presence of complete transport mixture lacking added ATP, creatine phosphatase or creatine phosphokinase, but supplemented with 50 U/ml apyrase and 1 mM CaCl_2_.

For the peptide competition experiments, SV40 TAg NLS peptide or ASV IN NLS peptide preparations were solubilized in a small amount of transport buffer and added directly to the complete transport mixture at the stated molar excess prior to the addition of the appropriate import substrate. For experiments with thioredoxin-integrase fusion protein (trx-IN(195–270)) as a competitor, the fusion protein was added in either 15 or 30-fold molar excess as indicated in the complete transport mixture, prior to the addition of import substrate to be assayed. For antibody inhibition experiments, digitonin permeabilized HeLa cells were either treated with 50 ug/ml antibody 3E9 against Impβ during the import reaction. More than 100 cells were counted for each experimental condition and the percentage of cells that had a mostly nuclear staining for the fusion protein was calculated. These competition and antibody experiments were repeated 3 times and representative data shown in Fig. 4.

For the Histone H1 competition experiments, permeabilized cell assays were done as above with 30 min incubation, but with the addition of a 15-fold molar excess of unlabeled Histone H1 in the complete transport mixture where indicated in Fig. 5. Histone H1 was labeled with Alexa Fluor 488 according to manufacturer's instructions (Molecular Probes) and its import assayed as with all other substrates. This experiment was repeated 3 times and representative data is shown.

### Construction of GST-fusion expression plasmids, preparation of GST-fusion proteins and cytosolic extract depletion experiments

Construction of the GST-Integrase fusion proteins was described previously [57]. A plasmid able to express the NLS of the U1A protein fused to GST was constructed by PCR of human cDNA with primers that amplified DNA encoding amino acids 94 to 204 of the U1A protein [35]. This DNA fragment was digested with BamH1 and EcoR1 and ligated into the GST expression plasmid pGEX-2TK (Amersham-Pharmacia). GST-M9 and GST-TAg were kindly provided by Gideon Dreyfuss and Michael Malim, respectively. All GST-fusion proteins were expressed and purified by the same methodology as previously described [57].

Cytosolic extract was depleted of factors which interact with specific NLS sequences as follows. Purified fusion proteins were mixed with glutathione-agarose beads (Sigma) and the amount of bound fusion protein was determined by SDS-PAGE. A standard volume (75 to 100 ul) of HeLa cytosolic extract was incubated with glutathione agarose beads normalized for the amount of fusion protein per bead volume (approximately 50–100 ug of fusion protein was used in a typical binding reaction). Extract and beads were incubated at 4°C for 1 hr with rocking. Beads were then pelleted at low speeds in a microfuge (4°C) and 25 ul of the resulting supernatant was utilized in the *in vitro *nuclear import assays.

## Conclusion

By use of an *in vitro *assay with digitonin-permeabilized cells, we confirmed that nuclear import of ASV IN is mediated by a previously identified NLS sequence. This import is active, saturable, ATP-dependent, and relies on cytosolic factors to transit through the nuclear pore complex. These results are consistent with a role for ASV IN in the nuclear import of the preintegration complex of this retrovirus.

Although the ASV NLS exhibits similarity to some classical NLSs, we present a variety of evidence that make it unlikely that the classical Impα/Impβ heterodimer is required for its import. The results indicate that the ASV IN NLS is recognized by other, perhaps Impβ-like soluble karyophilic protein(s), which is also able to mediate nuclear accumulation of the cellular linker histone H1. The system we describe may be useful to identify the factor(s) and evaluate its role in ASV replication.

## Abbreviations

IN:  integrase; NLS:  nuclear localization sequence; ASV:  avian sarcoma virus; HIV:  human immunodeficiency virus; BSA:  bovine serum albumin; WGA:  wheat germ agglutinin; GST: glutathione S-transferase.

## Competing interests

The authors declare that they have no competing interests.

## Authors' contributions

MDA: participated in the design and coordination of the study, performed or supervised all experiments with fusion substrates, and helped to write the final manuscript. MMS: participated in the design and execution of assays with the BSA substrates, and the writing of an original draft manuscript. AG: cloned and purified several NLS fusion proteins, and participated in the ASV NLS competition and mAb 3E9 inhibition experiments. KB: efforts were essential for refinement of the permeabilized cell assay, and preparation of new substrates and reagents tested. MH: purified NLS substrates and performed preliminary experiments on siRNA-mediated knockdown of Importins prior to infection experiments. AMS: participated in the design and coordination of this study, supervised its progress, and helped to write the final manuscript. All all authors read and approved the final manuscript.
